# Sleep as a mediator between chronic diseases and depression: a NHANES study (2005–2018)

**DOI:** 10.3389/fpsyg.2025.1522536

**Published:** 2025-01-29

**Authors:** Ming Tan, Haihong Zhao, Ruya Nie, Pingping Deng, Cuixiao Wang

**Affiliations:** ^1^Department of Otolaryngology-Head and Neck Surgery, Jingmen Central Hospital, Jingmen, Hubei, China; ^2^Department of Otolaryngology-Head and Neck Surgery, Jingmen Central Hospital Affiliated to Jingchu University of Technology, Jingmen, Hubei, China; ^3^School of Public Health, Guangxi Medical University, Nanning, Guangxi, China

**Keywords:** chronic disease, trouble sleeping, sleep hours, depression, mediation

## Abstract

**Objective:**

This article investigates the relationship between common chronic diseases and depression among US adults and examines the mediating role of sleep in this relationship, using a cross-sectional study to offer recommendations for depression prevention.

**Methods:**

This study analyzed data from 10,710 participants collected from the National Health and Nutrition Examination Survey (NHANES) between 2005 and 2018. Logistic regression, subgroup analysis, restricted cubic spline (RCS) analysis, and mediation analysis were employed to explore the relationship between common chronic diseases and depression, and the mediating role of sleep.

**Results:**

The adjusted model indicated that stroke (OR = 1.712, 95% CI: 1.399, 2.103), heart disease (OR = 1.419, 95% CI: 1.262, 1.598), diabetes (OR = 1.243, 95% CI: 1.116, 1.386), and hypertension (OR = 1.249, 95% CI: 1.160, 1.346) were associated with an increased probability of depression. Additionally, trouble sleeping (OR = 2.059, 95% CI: 1.790, 2.375) was associated with an increased probability of depression, while sleep hours (OR = 0.867, 95% CI: 0.846, 0.888) may decrease this probability. RCS analysis showed a non-linear relationship between sleep hours and the risk of depression. The final mediation analysis showed that trouble sleeping mediated 3.66% of the effect of stroke, 12.68% of heart disease, and 17.76% of diabetes on depression. Furthermore, trouble sleeping mediated 11.07% of the impact of hypertension on depression, while sleep hours mediated 5.36% of this impact.

**Conclusion:**

Chronic diseases and sleep problems may increase the likelihood of depression among U.S. adults, with sleep serving as a mediator between chronic diseases and depression.

## 1 Background

Depression is a widespread and severe mental disorder, characterized by symptoms including persistent sadness, reduced interest in activities, negative self-perception, and diminished self-esteem. Some patients have delusions of suspicion and victimization, pessimism, or worse, self-injury and suicidal tendencies (Mccarron et al., 2021), therefore, depression is also one of the main causes of disability and death. It has been reported that ~350 million people worldwide will experience cumulative depression in 2021 and that up to one million suicides will result from depression (Moore et al., 2022). The likelihood of suicide in individuals diagnosed with depression is ~50 times greater compared to that in the general population (Monroe and Harkness, 2022), leading to significant impacts on families and society.

Depression is not only a major contributor to global disability but also a significant factor in the global burden of disease (GBD 2019 Mental Disorder Collaborators, 2022), and thus requires substantial attention. Studying and identifying the contributing factors linked to the onset of depression is particularly crucial. The etiology of depression has been extensively investigated (Huang and Huang, 2023). Current evidence suggests that depression arises from a complex interplay of multiple factors, including biological mechanisms, environmental influences, genetic predisposition, and psychological components (Hammen, 2018). Additionally, sociodemographic factors such as marital status have also been identified as potential contributors to depressive symptoms (Bulloch et al., 2009). Evidence indicates that individuals with chronic diseases, such as cardiovascular and neurological diseases, exhibit a significantly elevated likelihood of experiencing depression in comparison to the general population. This includes conditions such as stroke, heart disease, diabetes mellitus, and hypertension (Golds et al., 2020). Research has shown a positive correlation between depression and an elevated prevalence of chronic illnesses (Hammen, 2018). The prolonged stress caused by certain chronic conditions can hinder individuals from fulfilling their life aspirations, significantly affecting mental wellbeing (Ronaldson et al., 2022).

Various elements may contribute to the relationship between chronic diseases and depression, including common genetic factors, convergent biological pathways, social factors, health behaviors, and psychological factors (Khawagi et al., 2024). People with chronic diseases frequently experience sleep problems (Liu et al., 2013; Zhong et al., 2020). Several studies have found that the illness and concerns about the future contribute to an increased prevalence of sleep abnormalities in these individuals (Golds et al., 2020). Existing evidence indicates that sleep disorders are closely associated with depression, with depressed patients often experiencing issues such as difficulty falling asleep, early awakening, and light sleep. Furthermore, sleep disorders are a risk factor for depression (Zhai et al., 2015; Huang and Huang, 2024; Zhong et al., 2022). Irregular sleep patterns and poor sleep quality have also significantly affected mood regulation and cognitive function (Furukawa et al., 2024).

In summary, chronic diseases and sleep problems both play significant roles in depression. We hypothesize that sleep may act as a mediating variable, influencing the effects of chronic diseases on depression. Consequently, this study seeks to examine the association between chronic illnesses and depression and the mediating effect of sleep among American adults, to provide a reference for the prevention and treatment of depression.

## 2 Methods

### 2.1 Study population

NHANES is a cross-sectional study implemented by the Centers for Disease Control and Prevention (CDC) on a biennial cycle. It employs a complex, stratified, multi-stage sampling methodology to ensure the sample is representative of the overall population within the United States (Qu et al., 2023). We used data from the NHANES 2005–2018 survey for this study. Participants with missing information on race, age, educational status, and smoking were excluded. Finally, 10,710 adults were included for analysis, as detailed in Supplementary Figure 1.

### 2.2 Assessment of depression

Depression was evaluated utilizing the Patient Health Questionnaire-9 (PHQ-9), a self-reported instrument grounded in the criteria outlined by the Diagnostic and Statistical Manual of Mental Disorders (DSM). The PHQ-9 assesses depressive symptoms that individuals have encountered during the preceding 2 weeks and consists of nine items scored on a 4-point scale. Scores were summed, with the total score for each participant ranging from 0 to 27. In this study, depression was characterized by a total PHQ-9 score of 10 or higher. This threshold is frequently employed in clinical and epidemiological research (Zimmerman, 2019).

### 2.3 Assessment of chronic diseases and sleep situation

Medical history and sleep duration were assessed based on the self-reported question or told by a doctor or other health professional.

### 2.4 Covariates

Covariates controlled for in this study included gender (male and female), age, BMI (kg/m^2^), race, income status based on the poverty impact ratio (PIR), smoking status (defined as having smoked at least 100 cigarettes in a lifetime), drinking status, education level, and marital status. Data for all covariates were obtained through direct measurements or standardized questionnaires.

### 2.5 Statistical analysis

In this study, R software version 4.3.0 was employed for data management and statistical analysis. Continuous variables were reported as mean ± standard deviation, with differences assessed using the independent samples *t*-test. Categorical variables were represented as frequencies and percentages, and a chi-square (χ^2^) test was utilized for univariate analysis. Influence factor analysis was performed using logistic regression models, with two models constructed: the crude model, which was unadjusted for variables, and the adjusted model, which controlled for all covariates. Subgroup analyses were conducted to further evaluate the impact of chronic diseases on depression. Interaction analyses were performed to examine the interactions between subgroup variables and chronic diseases, with RCS was conducted to examine the association between sleep duration and depression.

## 3 Results

### 3.1 Description of the study population

This analysis included a total of 10,710 adults ranging in age from 20 to 85 years, with a mean age of 46.13 ± 17.36 years. Among the participants, 301 (2.81%) had depression, 3,370 (31.5%) had hypertension, 1,023 (9.6%) had diabetes, 905 (8.5%) had heart disease, 304 (2.8%) had experienced a stroke, and 811 (7.6%) reported trouble sleeping (details are provided in Table 1).

**Table 1:** Baseline characteristics of study participants.

**Characteristics**	**Overall *N* (%)**	**No depression *N* (%)**	**Depression *N* (%)**	***P***
Age [mean (SD)]	46.13 (17.36)	46.23 (17.44)	42.53 (14.07)	< 0.001
Sex				< 0.001
Male	5,254 (49.1)	5,129 (49.3)	125 (41.5)	
Female	5,456 (50.9)	5,280 (50.7)	176 (58.5)	
Marital status				< 0.001
Single/divorced/widowed/separated	4,172 (39.0)	4,016 (38.6)	156 (51.8)	
Married/cohabited	6,538 (61.0)	6,393 (61.4)	145 (48.2)	
Race				0.186
White	5,579 (52.1)	5,434 (52.2)	145 (48.2)	
Black and others	5,131 (47.9)	4,975 (47.8)	156 (51.8)	
Education level				< 0.001
Below high school	2,131 (19.9)	2,056 (19.8)	75 (24.9)	
High school	2,472 (23.1)	2,387 (22.9)	85 (28.2)	
Above high school	6,107 (57.0)	5,966 (57.3)	141 (46.8)	
PIR				< 0.001
< 2	4,509 (42.1)	4,319 (41.5)	190 (63.1)	
≥2	6,201 (57.9)	6,090 (58.5)	111 (36.9)	
BMI (kg/m^2^) [mean (SD)]	28.87 (6.73)	28.82 (6.69)	30.54 (7.94)	< 0.001
Hypertension				< 0.001
No	7,340 (68.5)	7,167 (68.9)	173 (57.5)	
Yes	3,370 (31.5)	3,242 (31.1)	128 (42.5)	
Stroke				0.164
No	10,406 (97.2)	10,118 (97.2)	288 (95.7)	
Yes	304 (2.8)	291 (2.8)	13 (4.3)	
Heart disease				0.011
No	9,805 (91.5)	9,542 (91.7)	263 (87.4)	
Yes	905 (8.5)	867 (8.3)	38 (12.6)	
Diabetes				0.345
No	9,687 (90.4)	9,420 (90.5)	267 (88.7)	
Yes	1,023 (9.6)	989 (9.5)	34 (11.3)	
Drinking status				0.374
No	2,119 (19.8)	2,066 (19.8)	53 (17.6)	
Yes	8,591 (80.2)	8,343 (80.2)	248 (82.4)	
Smoking status				< 0.001
No	5,381 (50.2)	5,274 (50.7)	107 (35.5)	
Yes	5329 (49.8)	5135 (49.3)	194 (64.5)	
Sleephour [mean (SD)]	6.83 (1.34)	6.84 (1.33)	6.59 (1.56)	0.002
Sleep problem				< 0.001
No	9,899 (92.4)	9,646 (92.7)	253 (84.1)	
Yes	811 (7.6)	763 (7.3)	48 (15.9)	
Sleephour (hour)				0.004
< 7	4,181 (39.0)	4,038 (38.8)	143 (47.5)	
7-9	6,320 (59.0)	6,170 (59.3)	150 (49.8)	
>9	209 (2.0)	201 (1.9)	8 (2.7)	

### 3.2 Effect of chronic diseases on depression

#### 3.2.1 Logistic regression analysis results of the effect of chronic diseases on depression

**Figure 1:**
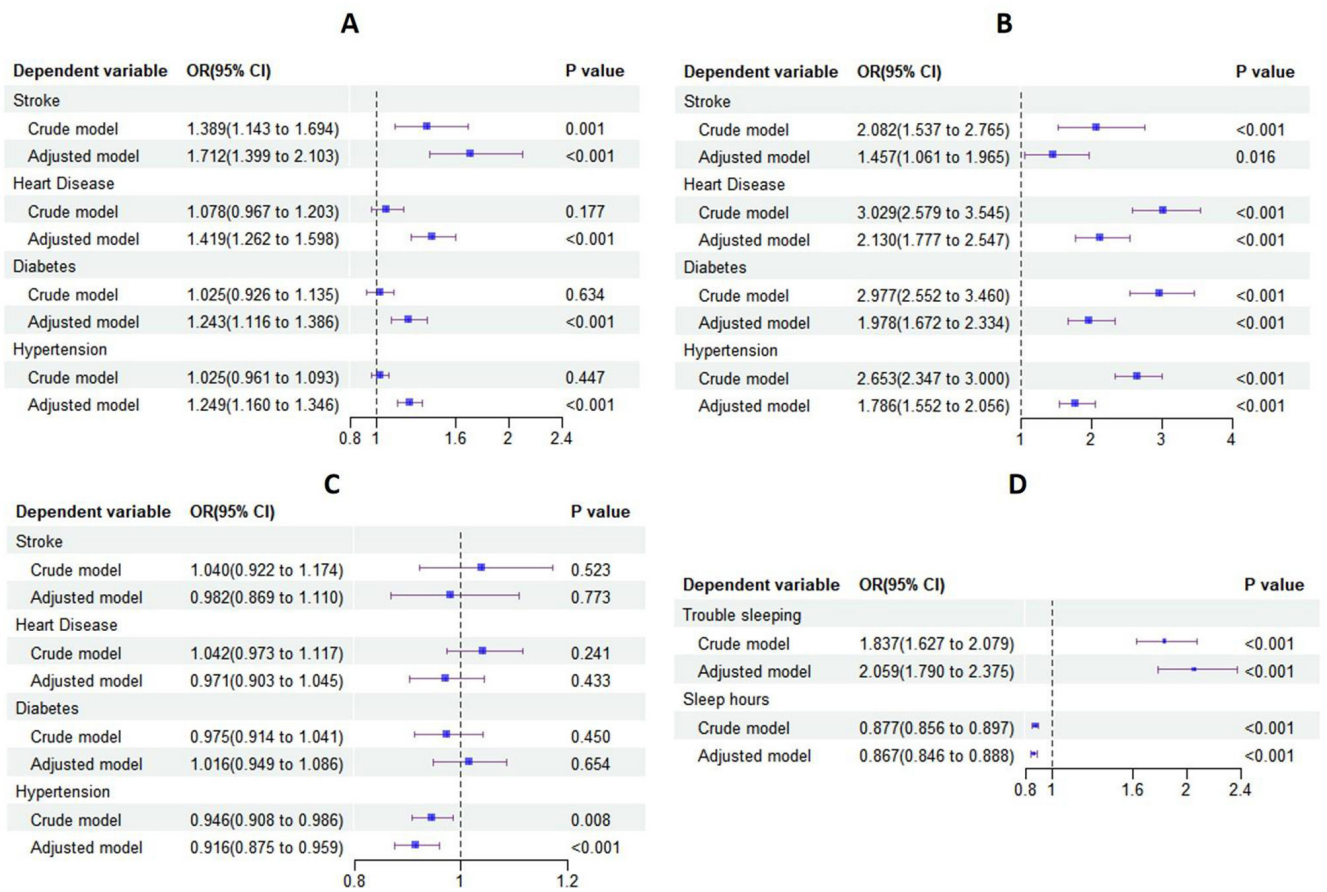
Forest plot of logistic regression results. **(A)** Forest plot of logistic regression results for the effect of chronic diseases on depression; **(B)** forest plot of logistic regression results for the effect of chronic diseases on trouble sleeping; **(C)** forest plot of logistic regression results for the effect of chronic diseases on sleep hours; **(D)** forest plot of logistic regression results for the effect of sleep on depression.

Logistic regression analysis was conducted with stroke, heart disease, diabetes, and hypertension as independent variables and depression as dependent variables, respectively. The adjusted model indicated that stroke (OR = 1.712, 95% CI: 1.399–2.103), heart disease (OR = 1.419, 95% CI: 1.262–1.598), diabetes (OR = 1.243, 95% CI: 1.116–1.386), and hypertension (OR = 1.249, 95% CI: 1.160–1.346) were all associated with an increased likelihood of depression, as shown in Figure 1A.

#### 3.2.2 Subgroup analysis results

Subgroup analyses were conducted based on age, sex, race, PIR, education level, marital status, smoking status, and drinking status. Logistic regression analysis was performed within each subgroup, with stroke, heart disease, diabetes, and hypertension as the independent variables and depression as the dependent variable. The results of these analyses generally supported the above findings. Additionally, there was a significant interaction between age and each of the chronic diseases in their effects on depression, as shown in Supplementary Figure 2.

### 3.3 Effects of chronic diseases on sleep

#### 3.3.1 Effect of chronic diseases on trouble sleeping

Stroke, heart disease, diabetes, and hypertension were used as independent variables, with trouble sleeping as the dependent variable in the logistic regression analysis. The adjusted model indicated that stroke (OR = 1.457, 95% CI: 1.061–1.965), heart disease (OR = 2.130, 95% CI: 1.777–2.547), diabetes (OR = 1.978, 95% CI: 1.672–2.334), and hypertension (OR = 1.786, 95% CI: 1.552–2.056) all had a significant effect on trouble sleep. These chronic diseases were associated with an increased likelihood of trouble sleeping, as shown in Figure 1B.

#### 3.3.2 Effect of chronic diseases on sleep hours

Logistic regression analysis was conducted with stroke, heart disease, diabetes, and hypertension as independent variables and sleep hours as the dependent variable. The adjusted model demonstrated a statistically significant effect of hypertension (OR = 0.916, 95% CI: 0.875–0.959) on sleep hours, as shown in Figure 1C.

### 3.4 Effect of sleep on depression

**Figure 2:**
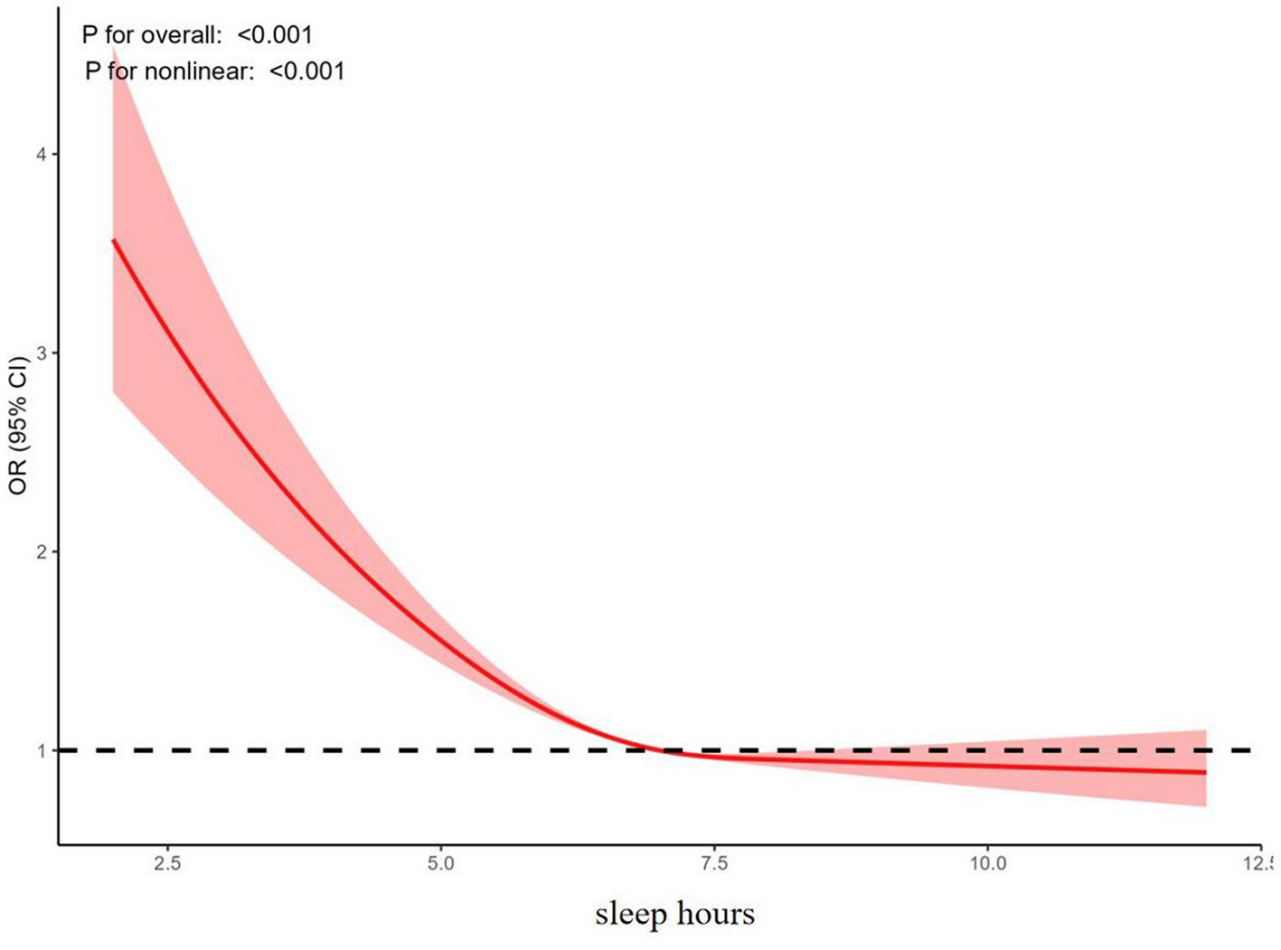
Results of RCS analysis of sleep hours and risk of depression.

Logistic regression analysis was conducted with trouble sleeping and sleep hours as independent variables and depression as the dependent variable. The adjusted model indicated that trouble sleeping (OR = 2.059, 95% CI: 1.790–2.375) might increase the likelihood of depression, while sleep hours (OR = 0.867, 95% CI: 0.846–0.888) may decrease the probability of depression, as shown in Figure 1D. Furthermore, the results of the RCS analysis revealed a nonlinear relationship between sleep hours and the risk of depression, as shown in Figure 2.

### 3.5 Mediation analysis

**Figure 3:**
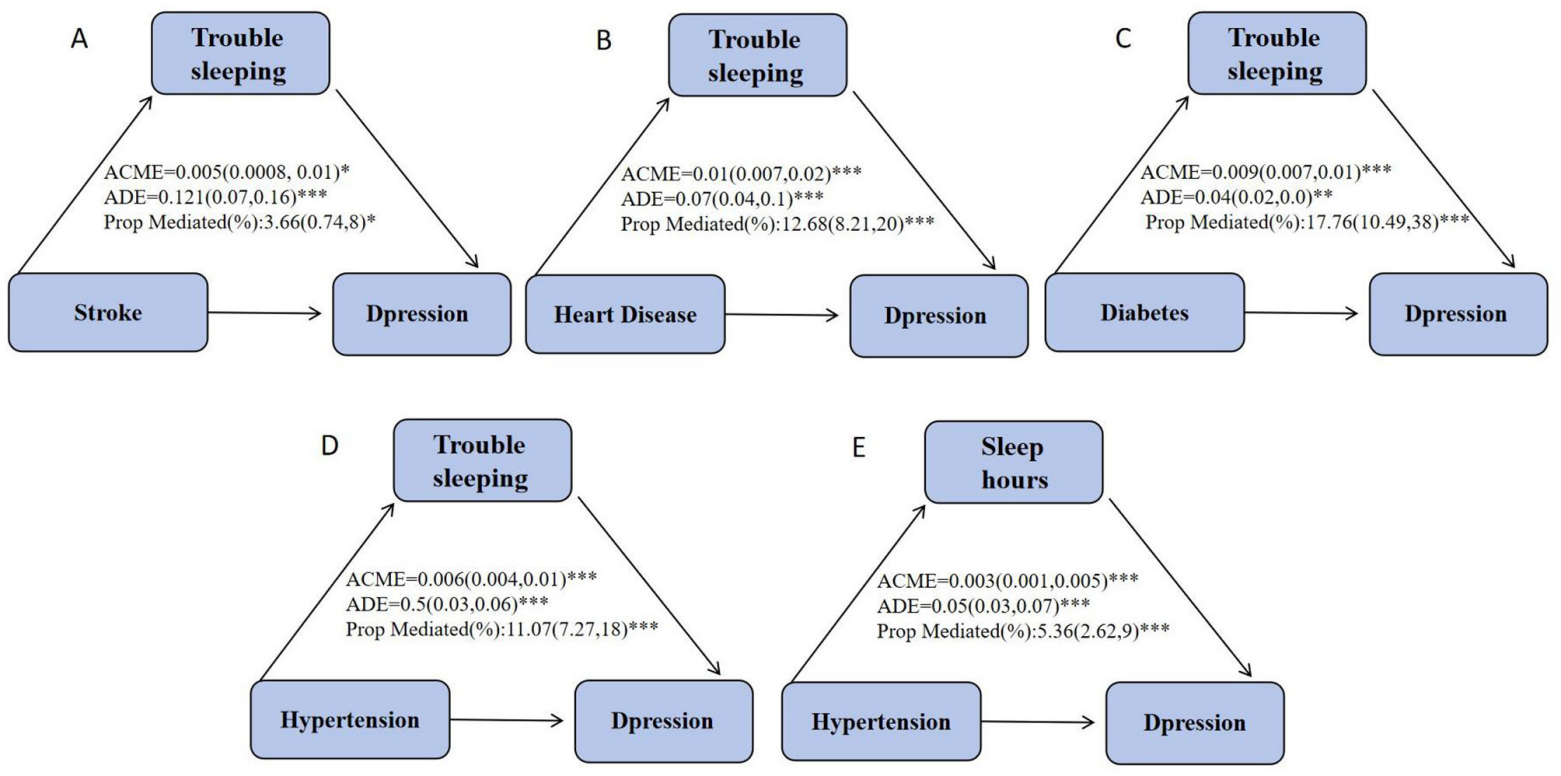
Estimated mediating effect of sleep duration on the association between chronic disease and depression. ACME, Average Causal Mediating Effect (indirect effect); ADE, Average Direct Effect. *p < 0.05, **p < 0.01, ***p < 0.001.

Mediation analysis was performed using the Bootstrap method, with each chronic disease as the independent variable, depression as the dependent variable, and trouble sleeping as the mediator. Similarly, mediation analysis was conducted with hypertension as the independent variable, depression as the dependent variable, and sleep hours as the mediator. The results indicated that trouble sleeping mediated 3.66% of the effect of stroke on depression, 12.68% of the effect of heart disease on depression, and 17.76% of the effect of diabetes on depression. Additionally, trouble sleeping mediated 11.07% of the effect of hypertension on depression, while sleep hours mediated 5.36% of the effect of hypertension on depression, as shown in Figure 3.

## 4 Discussion

In this study, we examined a total of 10,710 participants to investigate the relationship between chronic diseases and depression, as well as the mediating effect of sleep. The link between chronic diseases and depression is a significant topic in current medical research. Our findings suggest that heart disease, diabetes, hypertension, and stroke may increase the likelihood of depression, with sleep serving as a mediating factor. Evidence indicates that the prevalence of depression is notably higher among individuals with common chronic diseases, such as cardiovascular, cancer, metabolic, and neurological disorders, compared to the general population. Additionally, the risk of depression rises with the number of chronic diseases (Liu et al., 2023), and both a history of chronic diseases and the emergence of new chronic diseases contribute to this increased risk (Bayer et al., 2021). Depression further exacerbates the health impacts of chronic diseases, increasing both the burden of these conditions and the difficulty of their treatment (Golds et al., 2020). Madison's study also found that chronic diseases can intensify depressive symptoms (Sharp et al., 2021), and in severe cases, can even result in suicide in older adults (Zhang et al., 2018).

In addition to being associated with depression, our study found that heart disease, diabetes, hypertension, and stroke are linked to sleep disturbances, with hypertension also associated with reduced sleep duration. Sleep problems include sleep deprivation, altered sleep duration, and insomnia (You et al., 2023). A large-scale survey based on Pittsburgh Sleep Quality Index (PSQI) scores revealed a significant association between hypertension and poor sleep quality in rural China (Liu et al., 2015). Additionally, a Canadian community health survey indicated that nearly two-thirds of stroke patients had at least one sleep disorder, with the risk of developing a sleep disorder being 1.3–2.2 times higher than in the general population (Jeffers et al., 2023). The risk of sleep disorders remains elevated during the recovery period following a stroke. Furthermore, patients diagnosed with hypertension, heart failure, or atrial fibrillation are at a higher risk of developing sleep-breathing disorders (Cowie et al., 2021).

It is now recognized that sleep problems impact depression (Bampi et al., 2020). Adequate sleep regulates physiological, hormonal, and psychological processes and plays a crucial role in maintaining homeostasis within the body (Selvi et al., 2017). Both the quantity and quality of sleep change with age. Contrary to the long-held belief that sleep disorders are merely a consequence or incidental phenomenon of depression, recent evidence indicates that the relationship between sleep and depression is bidirectional (Ritter et al., 2015). Sleep disorders may also serve as potential risk factors for mental disorders. Studies have linked sleep disorders to frailty and cognitive decline (Taillard et al., 2021), and both frailty and cognitive impairment have been recognized as significant risk factors for depression. Additionally, prospective studies indicate that shortened sleep duration serves as an independent predictor of increased depressive symptoms, with sleep deprivation being a notable predictor of depression (Jansson-Fröjmark et al., 2016). A longitudinal study of Chinese college students (Wong et al., 2013) found that the duration of sleep was significantly correlated with follow-up depressive symptoms, with both short and long sleep durations being linked to an increased risk of depression in adults (Zhai et al., 2015). Depression scores were highly correlated with sleep duration (Wong et al., 2013). Evidence suggests that interventions targeting sleep, such as CBT-I and behavioral therapies, are effective in reducing depressive symptoms (Wong et al., 2013).

Our study found that chronic diseases can contribute to the development of depression by impacting sleep. Chronic diseases such as heart disease, diabetes, hypertension, and stroke can cause symptoms like pain, discomfort, and anxiety about health, which may lead to difficulties falling asleep, frequent awakenings, and reduced sleep quality. These sleep disturbances can further affect an individual's mood and increase the risk of depression (Ramos et al., 2023). Elevated blood glucose and dyslipidemia can promote depression by exacerbating inflammatory disorders and decreasing brain 5-HT3 levels. Sleep problems may mediate the relationship between chronic diseases and depression by influencing the body's stress response and inflammatory pathways (Khawagi et al., 2024). Chronic diseases often involve persistent inflammation, which can disrupt normal sleep patterns. Inflammatory cytokines such as TNF-α and IL-1β can alter sleep architecture and increase wakefulness during the night. This chronic inflammatory state not only affects sleep but also contributes to mood disorders like depression (Garbarino et al., 2021; Zheng et al., 2024). Additionally, sleep issues may reduce social interaction in patients with chronic diseases, affecting social support and potentially leading to depressive symptoms. Furthermore, sleep problems can impact cognitive function (Bergmans et al., 2019), as sleep deprivation affects attention, memory, and decision-making abilities (Taillard et al., 2021). This cognitive impairment can make it more challenging for individuals to manage daily life, thereby increasing the risk of depression.

We also observed an interaction between age and chronic disease in their effects on depression. One study found that the prevalence of depression varies across different age groups with chronic diseases, with individuals under 60 or 65 years of age being generally more prone to depression (Khaledi et al., 2019). Younger individuals with chronic diseases may experience increased pressure from occupational and family responsibilities, higher health expectations, and a lack of effective stress-coping strategies, which can hinder psychological adaptation (Moreno-Ligero et al., 2023). Consequently, they may be more susceptible to depression. Further research is needed to explore additional mechanisms underlying this interaction.

This study utilized data from the reliable and representative NHANES database, covering 2005–2018, with a sufficiently large sample size. Logistic regression, subgroup analysis, RCS, and mediation analysis were employed to assess the robustness of the results through multiple methods. However, this study has several limitations: first, being cross-sectional, it cannot establish causal relationships between chronic diseases, sleep, and depression; second, the data pertains to U.S. adults, so generalizing the findings to other countries requires further validation; and third, the study used subjective measures to assess sleep variables, which may not accurately reflect actual sleep situation. Additionally, potential confounding factors such as healthcare or comorbid mental health conditions were not identified in this study.

## 5 Conclusion

Chronic diseases and sleep problems may increase the likelihood of depression among U.S. adults, with sleep acting as a mediator between chronic diseases and depression. It is recommended that in future prevention of depression, controlling chronic diseases as well as addressing sleep may help reduce the probability of depression. Such interventions may include cognitive-behavioral therapy, medication, and health education. Improving sleep could mitigate the impact of chronic diseases on depression, thereby enhancing patients' quality of life and long-term health outcomes.

## Data Availability

The original contributions presented in the study are included in the article/Supplementary material, further inquiries can be directed to the corresponding author.
