# Supplementary material for: Sleep as a mediator between chronic diseases and depression: a NHANES study (2005–2018)
Source: Front Psychol. 2025 Jan 29;16:1522536. doi: 10.3389/fpsyg.2025.1522536 (PMC11814157; doi:10.3389/fpsyg.2025.1522536)

**Supplementary Figure**

Supplementary Figure S1: Flow chart of the population included in this study.


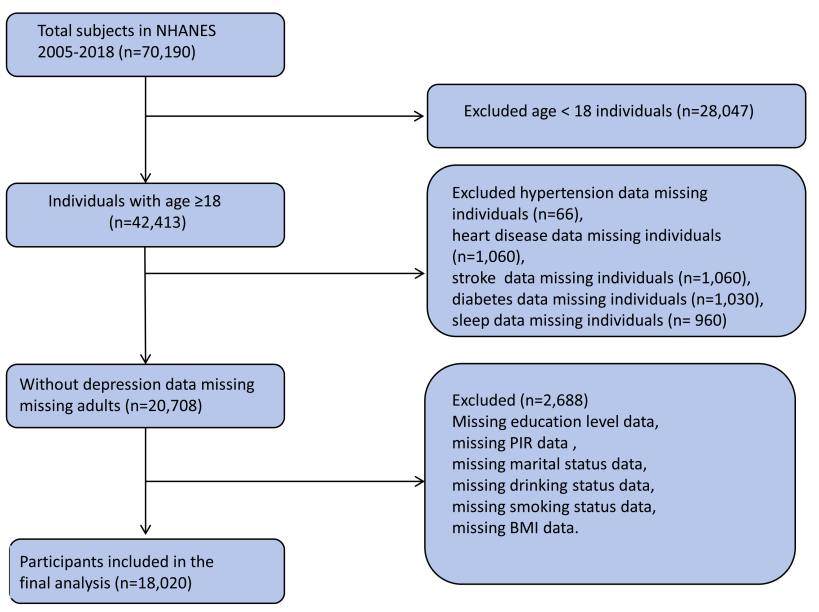


Supplementary Figure S2: Subgroup analysis results. A. Stroke, B. Heart disease, C. Diabetes, D. Hypertension.


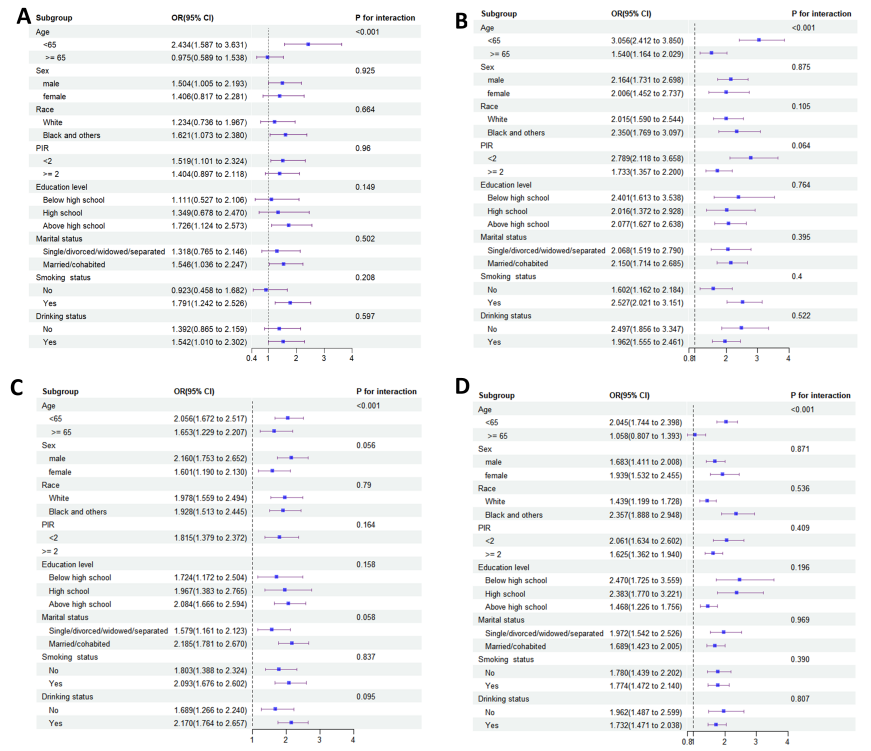

Supplement: Supplementary file 1 [file Data_Sheet_1.docx]
